# Maternal exposure to life events during pregnancy and congenital heart disease in offspring: a case-control study in a Chinese population

**DOI:** 10.1186/s12884-021-04154-0

**Published:** 2021-10-06

**Authors:** Jing Li, Yujiao Du, Yini Liu, Jiaoyang Du, Ruo Zhang, Pengfei Qu, Hong Yan, Duolao Wang, Shaonong Dang

**Affiliations:** 1 Department of Epidemiology and Biostatistics, School of Public Health, Xi’an Jiaotong University Health Science Center, Xi’an, 710061 Shaanxi Province China; 2 Department of Endocrinology, The Second Affiliated Hospital of Xi’an Jiaotong University, Xi’an, 710004 Shaanxi Province China; 3 Assisted Reproduction Center, Northwest Women’s and Children’s Hospital of Xi’an Jiaotong University, Xi’an, 710003 Shaanxi Province China; 4 Department of Clinical Sciences, Liverpool School of Tropical Medicine, Liverpool, UK

**Keywords:** Life events, Congenital heart disease, Case-control study

## Abstract

**Background:**

Previous studies have suggested that maternal stress could increase the risk of some adverse pregnancy outcomes, but evidence on congenital heart disease (CHD) is limited. We aimed to explore the association between maternal exposure to life events during pregnancy and CHD in offspring.

**Methods:**

The data was based on an unmatched case-control study about CHD conducted in Shaanxi province of China from 2014 to 2016. We included 2280 subjects, 699 in the case group and 1581 in the control group. The cases were infants or fetuses diagnosed with CHD, and the controls were infants without any birth defects. The life events were assessed by the *Life Events Scale for Pregnant Women*, and were divided into positive and negative events for synchronous analysis. A directed acyclic graph was drawn to screen the confounders. Logistic regression was employed to estimate the odds ratio and 95% confidence interval for the effects of life events on CHD.

**Results:**

After controlling for the potential confounders, the pregnant women experiencing the positive events during pregnancy had lower risk of CHD in offspring than those without positive events (OR = 0.38, 95%CI: 0.30 ~ 0.48). The risk of CHD in offspring could increase by 62% among the pregnant women experiencing the negative events compared to those without (OR = 1.62, 95%CI: 1.29 ~ 2.03). Both effects showed a certain dose-response association. Besides, the positive events could weaken the risk impact of negative events on CHD.

**Conclusion:**

It may suggest that maternal exposure to negative life events could increase the risk of CHD in offspring, while experiencing positive events could play a potential protective role.

**Supplementary Information:**

The online version contains supplementary material available at 10.1186/s12884-021-04154-0.

## Background

Congenital heart disease (CHD) is a kind of anatomical malformation caused by embryonic cardiovascular formation disorder or developmental abnormality. Statistically, about 4 to 13 per 1000 live births suffer from CHD around the world [1–3]. It is the most common type of birth defects, accounting for about 28% [1]. In China, the health burden caused by CHD is considered severe. According to the report on prevention and treatment of birth defects in China [4], the incidence was 4.95 per 1000 live births in 2011, and cases of CHD accounted for 26.7% of all monitored cases of birth defects. Due to the large population base of China, the total number of new CHD cases per year would be quite huge. It was estimated that there would be more than 130,000 new cases of CHD every year in China. Furthermore, CHD could easily cause pneumonia, severe hypoxia, cardiac failure and many other fatal complications and ultimately lead to infant death [5]. In addition, CHD can bring a massive economic burden, which was estimated to exceed 12.6 billion yuan in China each year [4].

Unfortunately, the exact etiology of CHD remains unclear, but it involves both genetic and environmental factors. Exploring the underlying etiologies, especially those preventable factors, is crucial for the primary prevention of CHD. Epidemiological studies have identified some related risk factors [6–10], such as sociological factors (age, residence, education et al.) and maternal exposure (infection, taking medicine, drinking, exposure to tobacco smoking, exposure to harmful substance, exposure to adverse environment and so on). Besides, folic acid supplementation has been confirmed as a protective factor in some studies [8, 10]. All these findings could provide scientific bases for the prevention and control of CHD.

As a preventable factor affecting pregnancy outcomes, the mental health of pregnant women has attracted more and more attention in recent years. It has been suggested that pregnant women exposed to stress were more likely to have adverse pregnancy outcomes [11–15], such as stillbirth, low birth weight, preterm birth, and small for gestational age. A few studies explored some types of birth defects but were almost conducted more than a decade ago. Hansen et al. discovered that maternal exposure to severe life events could cause cranial-neural-crest malformations [16]. Suarez et al. found that the occurrence of stressful life events was associated with risk of neural tube defects among Mexican-Americans [17]. Carmichael et al. had carried out three studies [18–20] in the past decade, all of which indicated that women exposed to stress or stressful life events during pregnancy probably had a higher risk of delivering infants with certain birth defects. However, the specific evidence on CHD remains limited. Considering the prevalence and serious consequences of CHD, it is extremely necessary to make efforts to explore the relation between maternal mental health and CHD further to provide more evidence for the prevention and control of CHD.

This study was devoted to explore the association between maternal experiencing life events during pregnancy and CHD in offspring. Life events experienced by pregnant women themselves could have an underlying impact on their mental health. The measure of life events in our study referred to a specialized scale [21], covering various aspects of events that pregnant women may experience during pregnancy. According to different effect of life events on the pregnant women, we divided them into positive and negative events for synchronous analysis.

## Methods

### Data and participants

The data in this study was based on an unmatched case-control study about CHD and its risk factors, which was conducted in Shaanxi province of China from January 2014 to December 2016. It was carried out in the six large hospitals responsible for monitoring birth defects in Xi’an, including First Affiliated Hospital of Xi’an Jiaotong University, Second Affiliated Hospital of Xi’an Jiaotong University, Xijing Hospital of the Fourth Military Medical University, Tangdu Hospital of the Fourth Military Medical University, People’s Hospital of Shaanxi Province and Northwest Women & Children’s Hospital. The cases were included according to the following inclusion criteria: the perinatal infants who were diagnosed with CHD according to the International Classification of Diseases-10 (ICD-10) from 28 weeks of gestation to 7 days after birth (including single live births and stillbirths), as well as the fetuses who were diagnosed with CHD by ultrasound examination in hospital less than 28 weeks of gestation. The controls were the newborn infants without any birth defects in corresponding hospitals. Briefly, a total of 2996 participants were recruited in the study. In this analysis, we excluded twins or multiple births, those diagnosed as other types of birth defects or CHD combined with other types of birth defects, those without a definite diagnosis, without a clear date of terminal pregnancy, unable to fill in a questionnaire and those lacked relevant variables data. Finally, 2280 participants were used as sample size for analysis after excluding those missing main variables or covariates. We conducted a post hoc analysis (G*Power program, version 3.1.9.4) [22] to assess the achieved power of our study. Based on our sample size and certain parameters of logistic regression in our analysis (OR = 1.64, α = 0.05, *R*^2^_for covariates_ = 0.21), the power about negative events was estimated at 99.7%. There was a similar power estimation conducted for positive events. It meant that the sample size included in our study could provide sufficient power to explore the association between exposure to life events (positive events and negative events) and CHD in offspring.

A self-designed structured questionnaire was used in the study to collect information, which has previously been published elsewhere [23]. The content included social demographic characteristics, maternal life behavior and environment during periconception, history of pregnancy and parturition, history of family disease, antenatal examination, disease and medication, nutrient supplementation, life events scale during pregnancy, neonatal information, etc. Maternal information was gathered by trained staff through face-to-face interviews with mothers, and newborn information was collected by the medical record system of hospital. The study was approved by the Human Research Ethics Committee of Xi’an Jiaotong University Health Science Center (No.2012008). All participants were aware of the content of study and signed the informed written consent before investigation.

### Assessment of maternal life events during pregnancy

The main variable life events were assessed by *Life Events Scale for Pregnant Women* (*LESPW*), a self-assessment scale compiled by Gao Yan et al. that has been published and widely used in China [21]. It included 53 representative life events, covering various changes that may occur in pregnant women’s daily life. A relevant study has suggested that the reliability and validity of *LESPW* could meet the requirement of statistical measurement (Cronbach’s alpha = 0.750) [24]. Unfortunately, these life events were not classified by their nature. Therefore, according to the potential influence of life events on pregnant women, we divided them into positive and negative events. Positive events (e.g., more care from family members) could have positive effect, and negative events could result in negative impact. Among all events, the description of 3 events ─ “house-moving”, “changing job-content or adjusting working hour and place” and “changing living habits such as sleep, diet and clothing” was equivocal (whether these changes were good or bad were unclear), so that it was difficult to determine the nature. Thus, the 3 events were excluded in our main analysis. We selected the remaining 50 life events, which included 9 positive events and 41 negative events (detail in Appendix 1). In addition, assuming that any changes may bring about stress on pregnant women, we regarded the 3 excluded events as negative events, i.e., 9 positive events and 44 negative events were included, for additional analysis to compare with the main analysis (Supplemental Table S1).

### Covariates

To clarify the association between life events and CHD more accurately, the relevant covariates should be considered for adjustment in analysis because they could confound the association. Reference to previous literature [6–10] as well as the characteristics of participants, there were 16 possible covariates concerned in total. They covered socio-demographic characteristics (maternal age, residence, and paternal education), maternal exposures (maternal drinking, exposure to tobacco smoke, exposure to harmful substance, exposure to adverse environment, folic acid supplementation, infection, depression, medicine-taking and prenatal examination) and history of childbearing (history of parturition, history of abortion, family history of CHD). In order to reduce unnecessary covariates and excessive adjustment, the directed acyclic graph (DAG) [25] (DAGitty program, version 2.3) [26] was adopted to screen the minimal sufficient set of covariates. DAG was introduced by Greenland et al. for causal analysis, and widely used in the field of epidemiology. It could visualize the causal relationship between variables, identifying the minimal sufficient adjustment sets to minimize confounding bias in epidemiological studies [25]. After analysis of DAG, 8 covariates were selected as adjusted variables, including maternal age (defined as < 25, 25 ~ 30 or > 30 y), residence (defined as urban or rural), maternal and paternal education (defined as ≥college or < college), history of parturition, history of abortion, infection during periconception, and abnormal prenatal examination (Figure S1). Considering that the genetic factor also played an important role in the occurrence of CHD, we additionally included the family history of CHD as potential covariate. Finally, a total of 9 covariates were included as adjusted variables in our analysis. Among them, history of abortion contained spontaneous abortion, induced abortion and medical abortion; infection during periconception period meant that pregnant women suffered from colds, fever, urogenital system infection and some viral infection from 3 months before pregnancy to the whole period of pregnancy; abnormal prenatal examination referred to abnormalities detected by B-mode ultrasound or fetal cardiac ultrasound examination before delivery.

### Statistical analysis

The categorical data was summarized by frequency and percentage, while the continuous data was summarized by mean ± standard deviation. The Chi-square (*χ*^*2*^) test and *t*-test were used to compare the differences between the case and control groups. Logistic regression models were constructed to estimate the odds ratio (OR) and 95% confidence interval (CI) for the effect of life events during pregnancy on CHD in offspring by not adjusting or adjusting for covariates. The adjusted covariates were 9 confounding factors as described above. Since both positive and negative life events occurred at the same time, we put them together in models for synchronous analysis. They were analyzed as both continuous variables and categorical variables classified by occurrence. For the latter, we also categorized them according to the frequency to explore the dose-response association. Moreover, based on the synthetical occurrence of positive and negative events, we created a new integrated variable, including four groups ─ “neither positive nor negative events group (P_0_N_0_)”, “only negative events group (P_0_N_1_)”, “only positive events group (P_1_N_0_)”, and “both of positive and negative events group (P_1_N_1_)”. Taking the P_0_N_0_ group as a reference, we calculated OR values of the other three groups. By comparing the OR values of the P_0_N_1_ and P_1_N_1_ groups, we aimed to explore the modifying effect of positive events under the circumstances that negative events occurred. In addition, subgroup analysis was employed to assess the consistency of the association across different categories of 8 selected covariates by DAG, and the remaining covariates were adjusted by multivariate logistic regression to estimate OR values of association between occurrence of life events (reference to non-occurrence) and CHD among each subgroup. Considering the smaller sample size for the family history of CHD group, we did not conduct subgroup analysis for this covariate. *P* < 0.05 was regarded as a statistical significance. SPSS version 22.0 (IBM SPSS Statistics, Chicago, IL, USA) was used for data analysis and R version 3.5.3 (R Development Core Team, Vienna, Austria) software was used to make plots.

## Results

### Characteristics of participants

**Fig. 1:**
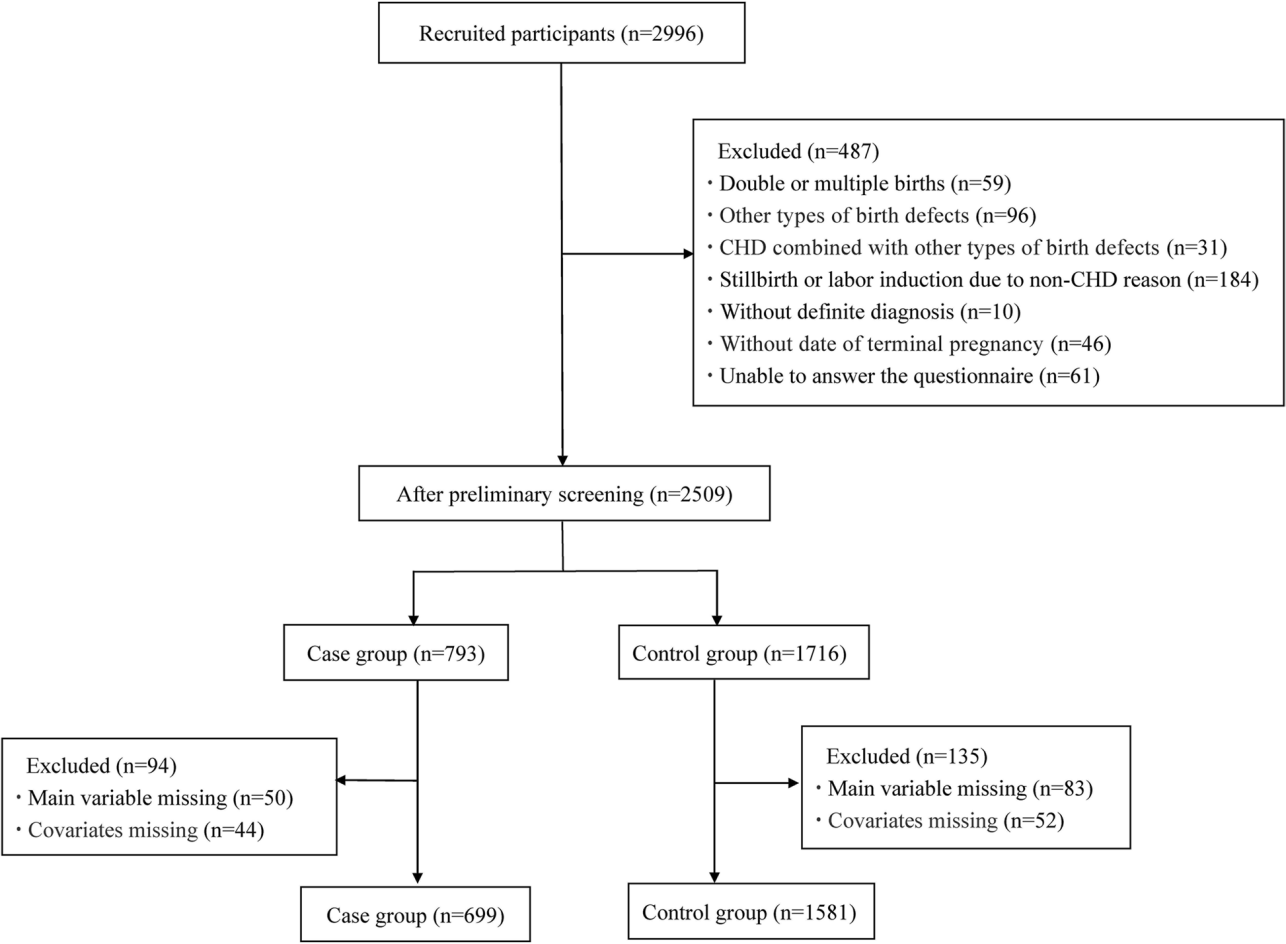
Flow chart of study participants

A total of 2280 subjects were included in this study, 699 in the case group and 1581 in the control group (Fig. 1). Table 1 showed the characteristics of pregnant women between the case and control groups. The differences between the two groups were statistically significant in maternal age, education, residence, history of parturition, infection during periconception and abnormal prenatal examination, while there was no significant difference in history of abortion.

**Table 1:** The characteristics of pregnant women between case and control group

Variables, n (%)	Case group	Control group	*χ*^*2*^ value	*p* value
	(*n* = 699)	(*n* = 1581)		
Maternal age				
<25 years	217 (31.04)	165 (10.44)	147.88	<0.001
25~30 years	350 (50.07)	1009 (63.82)		
>30 years	132 (18.89)	407 (25.74)		
Maternal residence				
Urban	237 (33.91)	1094 (69.20)	248.45	<0.001
Rural	462 (66.09)	487 (30.80)		
Maternal education				
≥ College	241 (34.48)	1237 (78.24)	407.12	<0.001
< College	458 (65.52)	344 (21.76)		
Paternal education				
≥ College	253 (36.19)	1250 (79.06)	396.51	<0.001
< College	446 (63.81)	331 (20.94)		
Family history of CHD				
No	1552 (98.17)	665 (95.14)	16.560	<0.001
Yes	29 (1.83)	34 (4.86)		
History of parturition				
No	410 (58.66)	1217 (76.98)	79.61	<0.001
Yes	289 (41.34)	364 (23.02)		
History of abortion				
No	422 (60.37)	1014 (64.14)	2.95	0.086
Yes	277 (39.63)	567 (35.86)		
Infection during periconception				
No	245 (35.05)	787 (49.78)	42.44	<0.001
Yes	454 (64.95)	794 (50.22)		
Abnormal prenatal examination				
No	549 (78.54)	1453 (91.90)	80.84	<0.001
Yes	150 (21.46)	128 (8.10)		
Positive events				
No	539 (77.11)	889 (56.23)	90.29	<0.001
Yes	160 (22.89)	692 (43.77)		
Negative events				
No	402 (57.51)	1029 (65.09)	11.90	<0.001
Yes	297 (42.49)	552 (34.91)		

### Association between maternal life events and CHD in offspring

As described in Table 2, the average number of positive life events in the case group was 0.30 ± 0.63, while 0.58 ± 0.79 in the control group; the average number of negative life events in the case group was 1.12 ± 2.06, while 0.72 ± 1.46 in the control group. After adjusting for the confounders, the risk of CHD in the offspring could be reduced by 48% (OR _*adj*_ = 0.52, 95%CI: 0.44 ~ 0.62) for each additional positive life event during pregnancy, while each additional negative life event occurring in pregnant women could make the risk of their offspring increase by 20% (OR _*adj*_ = 1.20, 95%CI: 1.13 ~ 1.28).

**Table 2:** The association between maternal life events and CHD in offspring

Life events	Case group	Control group	OR (95%CI) ^a^	
	(*n* = 699)	(*n* = 1581)	Unadjusted	Adjusted
Continuous variables, $\overline{x}\pm s$				
Positive events	0.30±0.63	0.58±0.79	0.46 (0.40, 0.54) ^**^	0.52 (0.43, 0.62) ^**^
Negative events	1.12±2.06	0.72±1.46	1.26 (1.19, 1.34) ^**^	1.20 (1.12, 1.28) ^**^
Categorical variables, n (%)				
Positive events				
No	539 (77.11)	889 (56.23)	1.00	1.00
Yes	160 (22.89)	692 (43.77)	0.34 (0.28, 0.42) ^**^	0.38 (0.30, 0.48) ^**^
Negative events				
No	402 (57.51)	1029 (65.09)	1.00	1.00
Yes	297 (42.49)	552 (34.91)	1.70 (1.40, 2.06) ^**^	1.62 (1.29, 2.03) ^**^

^a^Logistic model was used for estimating risk of life events with or without adjusting for covariates. Adjusted covariates included maternal age, residence, maternal education, paternal education, family history of CHD, history of parturition, history of abortion, infection during periconception, and abnormal prenatal examination

^*^ *p* < 0.01; ^**^ *p* < 0.001

According to the occurrence of life events, we analyzed them as binary variables. Among all participants, 852 pregnant women had ever experienced positive life events during pregnancy, 160 in the case group (22.89%) and 692 in the control group (43.77%); 849 pregnant women had experienced negative life events, 297 in the case group (42.49%) and 552 in the control group (34.91%). Both differences between the two groups had statistical significance. With confounders adjusted, the pregnant women with positive events experienced had a 62% lower risk of CHD in their offspring than those without (OR _*adj*_ = 0.38, 95%CI: 0.30 ~ 0.48). The pregnant women with negative events exposed were 1.62 times more likely to have CHD in their offspring than those without (OR _*adj*_ = 1.62, 95%CI:1.29 ~ 2.03).

### Dose-response association between frequency of life events and CHD in offspring

According to the frequency of life events, we investigated the dose-response association between them and CHD in offspring. As shown in Table 3, a trend of dose-response association was observed (*p*_*trend*_ < 0.001). For positive events, the odds of CHD in offspring decreased with the increase of event frequency. In contrast with those who had not experienced positive events, those experiencing one event had 63% lower odds, and those experiencing two or more events had 66% lower odds (OR_1*adj*_ = 0.37, 95%CI: 0.28 ~ 0.48; OR_2*adj*_ = 0.34, 95%CI: 0.21 ~ 0.53). For negative life events, the risk increased along with the accumulation of frequency. After adjusting for the confounders, the odds of CHD in offspring increased 1.40 times for the pregnant women experiencing 1 ~ 2 negative events (OR_1*adj*_ = 1.40, 95%CI: 1.09 ~ 1.79), 2.08 times for those with 3 ~ 4 events (OR_2*adj*_ = 2.08, 95%CI: 1.34 ~ 3.25) and 3.15 times for those with 5 or more events (OR_3*adj*_ = 3.15, 95%CI: 1.82 ~ 5.43) respectively, in comparison with those without negative events.

**Table 3:** The dose-response association between frequency of life events and CHD in offspring

Frequency	Case group	Control group	OR (95%CI) ^a^		*p* for trend
	(*n* = 699)	(*n* = 1581)	Unadjusted	Adjusted	
Positive events					
0	539 (77.11)	889 (56.23)	1.00	1.00	<0.001
1	121 (17.31)	524 (33.14)	0.35(0.27, 0.44) ^**^	0.37 (0.28, 0.48) ^**^	
≥2	39 (5.58)	168 (10.63)	0.26(0.17, 0.38) ^**^	0.34 (0.21, 0.53) ^**^	
Negative events					
0	402 (57.51)	1029 (65.09)	1.00	1.00	<0.001
1~2	196 (28.04)	422 (26.69)	1.42(1.15, 1.76) ^*^	1.40 (1.09, 1.79) ^*^	
3~4	55 (7.87)	84 (5.31)	2.32(1.58, 3.39) ^**^	2.08 (1.34, 3.25) ^*^	
≥5	46 (6.58)	46 (2.91)	4.36(2.73, 6.96) ^**^	3.15 (1.82, 5.43) ^**^	

^a^Logistic model was used for estimating risk of life events with or without adjusting for covariates. Adjusted covariates included maternal age, residence, maternal education, paternal education, family history of CHD, history of parturition, history of abortion, infection during periconception, and abnormal prenatal examination

^*^ *p* < 0.01; ^**^ *p* < 0.001

### Modification effect of positive life events on CHD in offspring

Further, we divided the variable into four integrated groups to explore modification of positive events: P_0_N_0_, P_0_N_1_, P_1_N_1_ and P_1_N_0_. Compared with the P_0_N_0_ group, the odds of CHD in the P_0_N_1_ group increased by 38% (OR _*adj*_ = 1.38, 95%CI: 1.05 ~ 1.82), while that in the P_1_N_1_ group decreased by 33% (OR _*adj*_ = 0.67, 95%CI: 0.49 ~ 0.91) (Table 4). It indicated that positive events could weaken or even modify the risk impact of negative events on CHD in offspring.

**Table 4:** The modified effect of positive events based on integrated grouping

Positive	Negative	Case group	Control group	OR (95%CI) ^a^	
Events	Events	(*n* = 699)	(*n* = 1581)	Unadjusted	Adjusted
0	0	346 (49.50)	649 (41.05)	1.00	1.00
0	1	193 (27.61)	240 (15.18)	1.51 (1.20, 1.90) ^†^	1.38 (1.05, 1.82) ^*^
1	1	104 (14.88)	312 (19.73)	0.63 (0.48, 0.81) ^†^	0.67 (0.49, 0.91) ^*^
1	0	56 (8.01)	380 (24.04)	0.28 (0.20, 0.38) ^†^	0.29 (0.21, 0.42) ^†^

^a^Logistic model was used for estimating risk of life events with or without adjusting for covariates. Adjusted covariates included maternal age, residence, maternal education, paternal education, family history of CHD, history of parturition, history of abortion, infection during periconception, and abnormal prenatal examination

^*^ *p* < 0.05; ^**^ *p* < 0.01; ^†^ *p* < 0.001

### Subgroup analysis

**Fig. 2:**
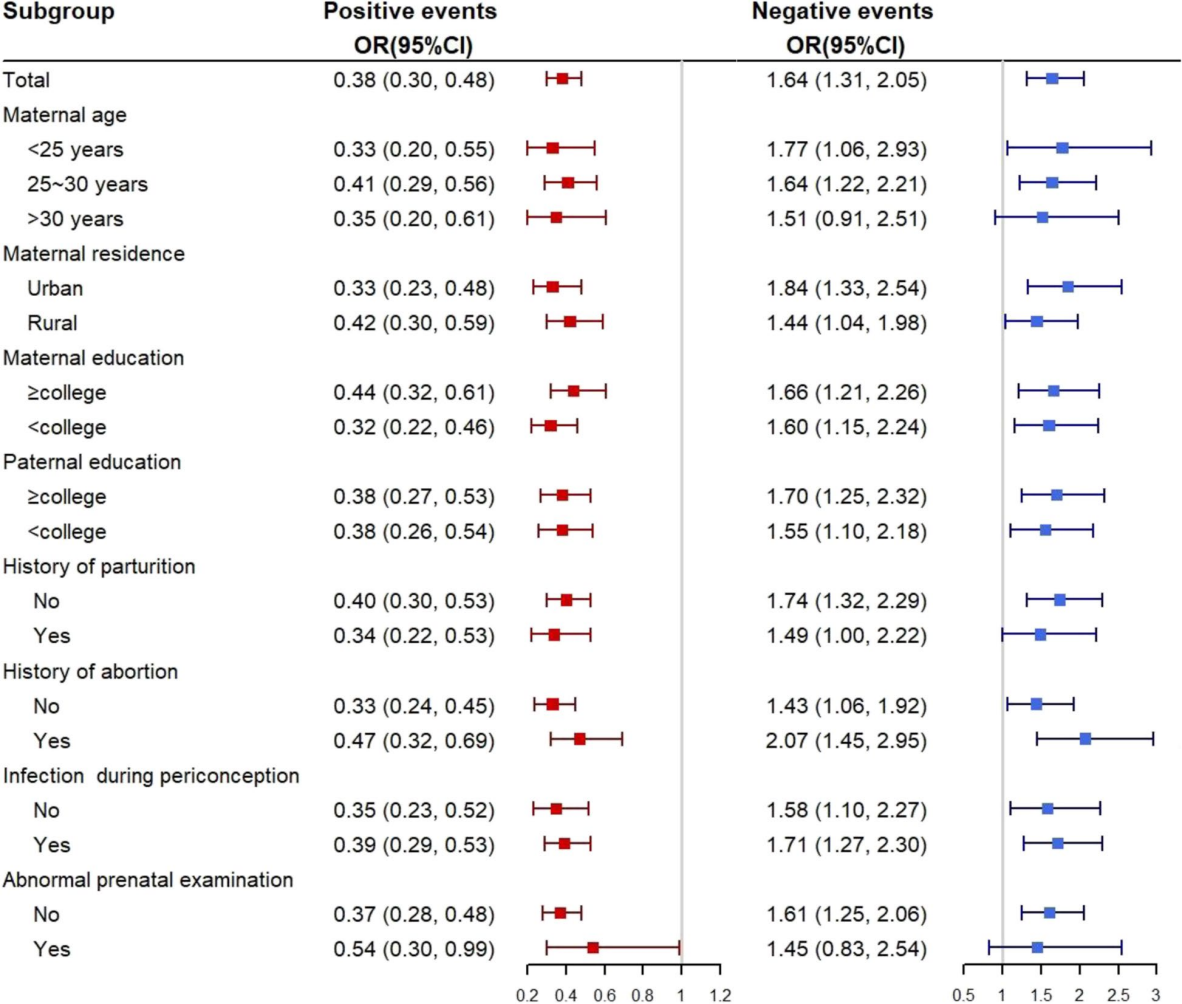
Subgroup analysis of association between occurrence of life events and CHD. The ORs were estimated by multivariate logistic regression with the remaining covariates except the subgroup covariate adjusted. ^*^ *p* < 0.05; ^**^ *p* < 0.01; ^***^ *p* < 0.001

The adjusted ORs of association between occurrence of positive or negative life events and CHD in offspring by selected covariates were shown in Fig. 2. The protective effects of positive events among pregnant women in each subgroup were observed significantly, demonstrating well stability and consistency of the association between life events and CHD. For negative events, except for two subgroups of those older than 30 years and those with an abnormal prenatal examination, the significant risk of negative events for CHD was found in the rest of subgroups, implying an overall robust association.

In addition, as mentioned above, 3 excluded events about life or work changes were supposed to be regarded as negative events for additional analysis. As shown in Supplemental Table S1, pregnant women with positive events experienced had 60% lower odds of CHD in offspring than those without (OR _*adj*_ = 0.40, 95%CI: 0.31 ~ 0.51), while pregnant women with negative events experienced had 21% higher odds than those without (OR _*adj*_ = 1.21, 95%CI: 0.97 ~ 1.51). Compared with the results of the primary analysis, the adjusted risk effect of negative events was reduced and not statistically significant, but the direction remained the same as before.

## Discussion

With the highest incidence of birth defects, CHD had a serious adverse impact on health and quality of life in offspring [27]. Life events, as common and modifiable factors, played an important role in the prevention of CHD in offspring. As we know, there has been a lack of studies specialized in the association between life events and CHD so far. Besides, most previous studies as described above focused on the adverse influence of negative or stressful events on offspring, failing to take positive events into account. It was one-sided to only assess the impact of negative events, because positive events (such as more care from family members) could play a potential buffer and support role when coping with the stress reaction of negative events [20]. Therefore, based on *LESPW*, we made comprehensive analysis of effect of both positive and negative life events during pregnancy on CHD in offspring. After adjusting for confounding factors, it was found that the experience of positive events probably reduced the risk of CHD in offspring, while the experience of negative events was likely to increase the risk. Besides, the dose-response association was found about effect of life events, that is, both the protective effect of positive events and the risk effect of negative events tended to increase with the frequency of events. Further, under the circumstances that the negative events had occurred, the positive events could modify their negative impact on CHD to some extent.

Previous relevant studies have suggested that negative or stressful life events could lead to higher risk of some birth defects [16–20, 28, 29]. However, most assessments of negative or stressful events were incomprehensive. Hansen et al. defined serious life events as partners’ or children’s death or first hospital admission for cancer or acute myocardial infarction [16]. Suarez et al. considered residential & occupational histories as well as any accidents or injuries to measure maternal life-event stress [17]. Carmichael et al. conducted three studies based on different generations of population, whose stress exposure variable included 3 events [18], 18 events [19] and 7events [20], respectively; two other studies just investigated the stress from natural disasters [28, 29]. Compared with these studies, the assessment in our study contained more comprehensive and representative events. Similar to previous findings, we found the risk impact of negative life events on CHD in offspring. As for positive events, only two studies considered the role of social support. One study found that social support was associated with reduced risks of some birth defects [20], while the other failed to found this potential effect [17]. In our study, positive events included 9 events (representing the family and social support) that had positive impacts on pregnant women, and protective effect on CHD was discovered. Additionally, in terms of the degree of effect, the protective effect of positive events seemed to be stronger than the risk effect of negative events. We further found that positive events had modification effect to some extent, which could modify the risk impact of negative events on CHD.

As a special group, pregnant women may experience various life events during the whole period of pregnancy. Among them, negative events could reflect the stress on pregnant women to some extent [30]. Although the mechanism of prenatal stress on fetal development was unclear, some studies suggested that it may be related to the hypothalamic-pituitary-adrenocortical axis (HPA-axis) and relevant regulatory factors [31–33]. Maternal prenatal stress may have programming effects on the physiological development of their offspring by producing abnormal activities of the maternal HPA-axis [31]. Stress could lead to excessive production of maternal glucocorticoid, which may stimulate the production of placental corticotropin releasing hormone (CRH), activate the HPA-axis of offspring, and consequently has adverse effect on growth and development of fetus [33–35]. Early animal experimental studies have suggested that injecting glucocorticoid into pregnant mice could result in cleft palate and other congenital malformations in their offspring [36, 37]. In addition, some epidemiological studies have also found that the use of glucocorticoids during pregnancy may slightly increase the risk of birth defects [38, 39]. By contrast, positive events could enable pregnant women to gain social and emotional support, playing a potential protective role [40]. Pluess et al. found positive life events may predict lower morning cortisol levels in pregnant women [40]; Steptoe et al. also found that positive emotions could reduce cortisol levels during the daytime [41]. From this point of view, positive events may contribute to reducing adverse endocrine changes of stress response brought by negative events, acting as a kind of potential buffer against the adverse impact of negative events and thus playing a protective role.

This study was based on case-control data for CHD with a large sample size. The main variable as life events were measured by a specialized scale with all-round events, and classified by positive or negative effects for comprehensive analysis. The necessary confounders were screened by DAG to make the adjustment more appropriate. Besides, we analyzed the main variable from different perspectives to make the results more robust. Nonetheless, there were still some limitations in our study. Firstly, different subtypes of CHD occurred at different embryonic development stages: some of them included single atrium, single ventricle and transposition of the great arteries occurred in the early embryonic stage, while patent foramen ovale and patent ductus arteriosus occurred after delivery. Unfortunately, considering smaller sample size for the subtypes, we didn’t analyze specific subtypes but only overall CHD. Therefore, further study on life events and subtype of CHD would be required. Secondly, the recall bias in life events was inevitable. On the one hand, the information was collected retrospectively; on the other hand, women may be unwilling to recall or mention certain negative events. Thirdly, we asked the participants to recall life events during overall period of pregnancy in this study. It was difficult to distinguish exact occurrence time of life events so that we failed to rule out some individuals who may experience life events after CHD had formed, which was another limitation. Even though, our study still suggested a possibility that the exposure to adverse life events during could be related to increasing risk of overall CHD. However, further investigation on occurrence of life events is required by means of prospective study. Fourthly, there may still be some potential confounders failing to be collected and adjusted. Finally, the causal relationship was unable to be determined due to the nature of case-control study design.

## Conclusion

This study suggested that negative events during pregnancy could be the risk factor of CHD in offspring while positive events were the protective factor. Efforts should be made to initiate a psychological intervention for pregnant women, which not only tries to reduce maternal stress or negative stimulation during pregnancy, but also provides more support or care to increase their positive experience. Thus, the improvement of mental health would contribute to the prevention and control of CHD in offspring.

## Supplementary Information

**Additional file 1: Figure S1.** Directed acyclic graph (DAG) in our study. **Table S1.** Association between maternal exposure to life events and CHD in offspring (including all 9 positive events and 44 negative events). **Appendix 1.** The detailed content of life events

## Data Availability

The datasets used and analyzed during the current study are available from the corresponding author on reasonable request.

## Abbreviations

CHD: Congenital heart disease
LESPW: Life Events Scale for Pregnant Women
OR: Odds ratio
