# Supplementary material for: Maternal exposure to life events during pregnancy and congenital heart disease in offspring: a case-control study in a Chinese population
Source: BMC Pregnancy Childbirth. 2021 Oct 6;21:677. doi: 10.1186/s12884-021-04154-0 (PMC8496089; doi:10.1186/s12884-021-04154-0)
Supplement: Supplementary file 1 — Additional file 1: Figure S1. Directed acyclic graph (DAG) in our study. Table S1. Association between maternal exposure to life events and CHD in offspring (including all 9 positive events and 44 negative events). Appendix 1. The detailed content of life events [file 12884_2021_4154_MOESM1_ESM.docx]

**Fig S1.** Directed acyclic graph (DAG) in our study

Reference to previous literatures and characteristics of our data, 16 possible covariates were included in DAG as shown below. By means of DAG analysis, the minimal sufficient set of covariates contained 8 covariates: maternal age, residence, maternal and paternal education, history of parturition, history of abortion, infection during periconception, and abnormal prenatal examination. They were regarded as adjusted variables in analysis of association of CHD in offspring with maternal life events.


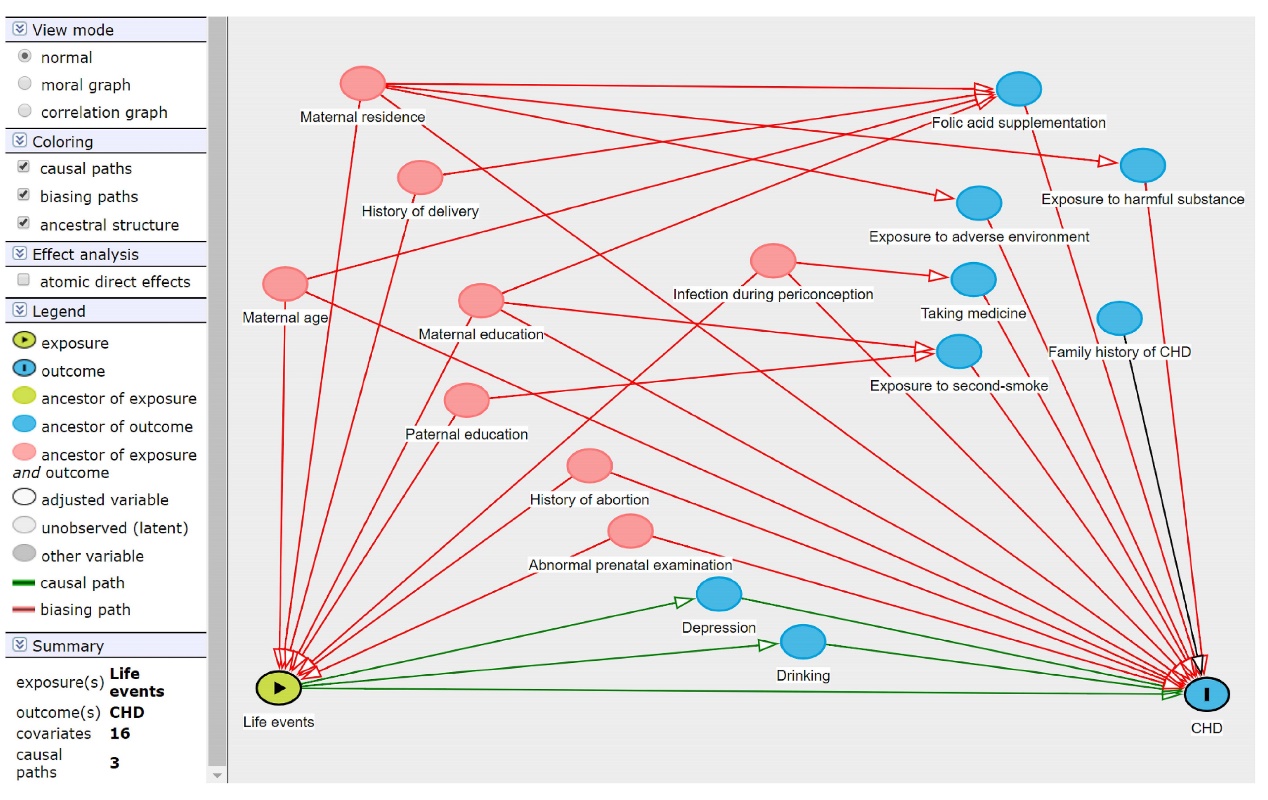


**Table S1.** Association between maternal exposure to life events and CHD in offspring (including all 9 positive events and 44 negative events)

| Life events | Case group | Control group | OR (95%CI) | |
| --- | --- | --- | --- | --- |
|  | (n=699) | (n=1581) | Unadjusted | Adjusted ^a^ |
| Positive events | | | | |
| No | 539 (77.11) | 889 (56.23) | 1.00 | 1.00 |
| Yes | 160 (22.89) | 692 (43.77) | 0.36 (0.29, 0.44) | 0.40 (0.31, 0.51) |
| Negative events ^b^ | | | | |
| No | 372 (53.22) | 838 (53.00) | 1.00 | 1.00 |
| Yes | 327 (46.78) | 743 (47.00) | 1.26 (1.05, 1.53) | 1.21 (0.97, 1.51) |

^a^ Adjusting maternal age, residence, maternal education, paternal education, family history of CHD, history of parturition, history of abortion, infection during periconception, and abnormal prenatal examination

^b^ Three events with equivocal nature mentioned above were regarded as the negative life events, that is, negative events contained 44 events

**Appendix 1** The detailed content of life events

| Positive life events | | | |
| --- | --- | --- | --- |
| 1 | Got more care from family members | 2 | Got help and care from someone else besides family members |
| 3 | Improved the relationship with husband | 4 | Improved the strained relationship with other family members (except husband) |
| 5 | Got rewards and improvements | 6 | Husband got rewards and improvements |
| 7 | Achieved outstanding individual results | 8 | Lightened the workload |
| 9 | Family and personal income increased significantly |  |  |
| Negative life events | | | |
| 1 | Suffered from injury or illness | 2 | Family members were injury or illness |
| 3 | Suffered from scare | 4 | Felt troubled inexplicably sometimes |
| 5 | Misunderstood with parents | 6 | Poor relationship with other family members |
| 7 | Quarreled with others for life trifles | 8 | Poor relationship with mother-in-law |
| 9 | Poor relationship with neighbors | 10 | Poor relationship with colleagues |
| 11 | Family financial difficulties | 12 | Medium debt |
| 13 | Something stolen or property loss | 14 | Got involved in legal disputes |
| 15 | Being misunderstood, discriminated or talked | 16 | Family members was subject to criminal sanctions |
| 17 | High pressure from work or study | 18 | Being dissatisfied with current work |
| 19 | Worried that work or study was affected by pregnancy | 20 | Suffered from accident or natural disaster |
| 21 | Deducted bonuses, fines or being punished | 22 | Being laid off |
| 23 | Husband had a rough time at work | 24 | Husband was laid off |
| 25 | Separation in two places (due to work) | 26 | Separation (due to emotional problem) |
| 27 | Divorce | 28 | Suffered from violence form husband |
| 29 | Found husband had an extramarital affair | 30 | Found husband suffered from sexually transmitted diseases |
| 31 | Found someone reliable lied to you | 32 | Lied to someone else |
| 33 | Break with friends | 34 | Friends died |
| 35 | Husband died | 36 | Family members died (except husband) |
| 37 | Abortion for several times or infertility for several years | 38 | Worried the fetal development was affected by work (such as noise, radiation, etc.) |
| 39 | Abnormal fetal development found by examination | 40 | Worried about dissatisfaction with the child's gender |
| 41 | Disagreement on delivery mode between family members |  |  |
| Indefinable life events | | | |
| 1 | house-moving | 2 | changed job-content or adjusted working hour and place |
| 3 | changed living habits such as sleep, diet and clothing |  |  |
